# Neuro-Behçet’s disease in childhood: A focus on the neuro-ophthalmological features

**DOI:** 10.1186/1750-1172-8-18

**Published:** 2013-01-29

**Authors:** Paolo Mora, Chiara Menozzi, Jelka G Orsoni, Pierangela Rubino, Livia Ruffini, Arturo Carta

**Affiliations:** 1 Institute of Ophthalmology – Department of Biological, Biotechnological, and Translational Sciences, University of Parma, via Gramsci 14, 43126, Parma, Italy; 2 Nuclear Medicine Department – Azienda Ospedaliero, Universitaria of Parma, Parma, Italy

## Abstract

Neuro-Behçet’s disease (NBD) involves the central nervous system; peripheral nervous system involvement is not often reported. NBD is quite common in adult patients and occurs rarely during childhood and adolescence. Young patients may share symptoms and signs of NBD with other neuro-ophthalmological disorders (e.g. idiopathic intracranial hypertension); thus, making the differential diagnosis difficult. Neuroimaging is mandatory and necessary for a correct NBD diagnosis but in children radiological examinations are often difficult to perform without sedation. From 1971 to 2011, 130 patients aged ≤16 years have been reported with NBD, according to retrospective surveys, case series, and case reports. The origin of the reported cases met the well-known geographical distribution of Behçet’s disease (BD); the mean age at presentation of neurological findings was 11.8 years, with male gender prevalence (ratio, 2.9:1). We considered in detail the neuro-ophthalmological features of the 53 cases whose neuroimaging alterations were described with an assigned radiological pattern of the disease (parenchymal: 14 cases, non-parechymal: 35 cases, and mixed: 4 cases). In 19/53 patients (36%), neuro-ophthalmological symptoms anticipated any pathognomonic sign for a BD diagnosis, or only occasional aphtae were recalled by the patients. Family history was positive in 17% of subjects. Headache was reported in 75% of the patients; in those presenting with cerebral vascular involvement, headache was combined to other symptoms of intracranial hypertension. Papilledema was the most frequently reported ophthalmological finding, followed by posterior uveitis. Treatment consisted of systemic steroids in 93% of patients, often combined with other immunosuppressive drugs (especially colchicine and azathioprine). Clinical recovery or improvement was documented in the large majority of patients. Nine subjects had definitive alterations, and one died. Based on our review and personal experience, a delayed diagnosis, and the consequently delayed immunosuppressive treatment, may favour permanent sequelae, in particular, optic atrophy.

## Introduction

Behçet’s disease (BD) is an inflammatory, multisystem disease named after the Turkish dermatologist who first correlated the three characteristic findings, including recurrent oral and genital aphtae, and uveitis [1]. The currently addressed diagnostic criteria were defined in 1990 by the International Society for Behçet’s Disease [2]. However, these criteria make no specific reference to central nervous system (CNS) involvement. This condition, usually called neuro-Behçet’s disease (NBD), has variable prevalence depending on the series, but can represent 9–10% of affected patients [3]. Cerebral computed tomography (CT), magnetic resonance imaging (MRI), magnetic resonance angiography (MRA), and functional neuroimaging such as single-photon emission computed tomography (SPECT) have become the reference radiological tools for diagnosis and have proven particularly useful in ruling out differential diagnoses (e.g., idiopathic intracranial hypertension, IIH). The radiological pattern of the disease has been divided into two major forms: 1) the parenchymal form, which is clinically subdivided into acute and chronic progressive; and 2) the non-parenchymal (or vascular) form [4]. In the parenchymal type, the acute pattern is characterised by acute meningoencephalitis with or without focal lesions; the chronic progressive pattern is characterised by slowly progressive central and peripheral alterations such as dementia, ataxia, or dysarthria, along with a persistently marked elevation of interleukin-6 in the cerebrospinal fluid [5-7]. In the non-parenchymal type, parenchymal damage is possible, but occurs secondary to pathological processes localised in the large venous or, more rarely, arterial cerebral vessels [8,9]. The concomitance of parenchymal and vascular involvement has been classified in some studies as mixed parenchymal and non-parenchymal [3,10].

Unlike younger subjects, adult patients often develop neurological involvement after some non-neurological manifestations of BD [11], which greatly favours a correct diagnosis and early treatment. Although not predominant, neuro-ophthalmological findings in BD have been reported for a long time in children, and optic atrophy is a major consequence [12-14]. However, the prevalence of such complications is difficult to ascertain from available reports, mainly because of the limited number of patients and the lack of clear details about respective neurological findings [3]. An extensive review of the literature on pediatric NBD, focusing on the neuro-ophthalmological features in cases with a definitive radiological diagnosis, is presented and discussed.

## Methods

This review was designed to examine all reports concerning subjects aged ≤16 years having BD and presenting with any related CNS involvement. A diagnosis of BD was considered if it matched the international classification criteria (Japanese or European) [2,15]. Other criteria were accepted when they were the standard by the date of publication or when they were based on distinctive neuroimaging.

We searched MEDLINE (October 1971–December 2011) and EMBASE (January 1980–December 2011) singly and in combination using the following key words: Behçet, Behçet’s disease, neuro-Behçet, neurology, imaging, venous thrombosis, central nervous system, optic nerve, paediatric, juvenile, and young. Articles written in English, French, German, and Italian were fully reviewed. Other languages were reviewed from the Abstract. The Reference sections of the articles were reviewed for any article that had been missed in the electronic search.

The primary outcome of this review was to evaluate the neurological and ophthalmological features of the disease in young patients. Secondary outcomes that were evaluated were other aspects of the disease, in particular: time of presentation of neuro-ophthalmological findings, family history, drugs used for treatment, and prognosis. Considering the difficulty of the differential diagnosis and the problems related to classifying NBD, we decided to limit a detailed analysis of the above mentioned outcomes to those cases with a definitive diagnosis based on neuroimaging. A concise overview of the same parameters was however performed in cases for whom a precise radiological characterisation was not reported. The demographic data were considered for all patients described in the literature.

## Results

### Source literature

In total, 38 articles have reported 130 patients within the considered range of age who were affected by BD with neurological involvement (excluding the cases with isolated headache) [12,14,16-51]. Among these articles, two were nationwide retrospective surveys [31,32], and five studies described eligible subjects selected from large retrospective chart reviews [14,30,43,48,49]. In one study, new examinations were performed in a small cohort of subjects [34]. The remaining studies were single-case reports or case series.

### Demographics

The demographics of all described subjects are summarized in Table 1. Sixty-nine patients (53%) originated from the so-called “silk road” stretching from Morocco to Japan [14,17,18,28,30-33,35,37-42,44,48-50]. The large majority of these subjects (64/69 subjects) were from Western Asia, and only five were from other regions along this historical route [31,41]. In addition to the large number of Asian subjects, 24 patients (26%) were from Europe: 14 were French [12,32,36,48], eight were Italian [29,34,46,47], and two were from the UK [22,43]. The remaining geographical areas had minor prevalence (see Table 1). [16,19-21,23-27,37,45] The origins of 25 patients were not specified. Gender was reported in 71/130 subjects, with 53 males (75%) and 18 females. The mean age calculated from the available data was 11.8 years.

**Table 1:** Demographics of patients with juvenile Neuro-Behçet disease reported in the literature from 1971 to 2011 (only when specified)

**Origin**	**No. of Patient**	**Male/Female**	**Age (median; range)**
West Asia	64	37/10	13; 10–15
Europe	24	6/2	12; 8–15
North America	8	4/4	11; 1–16
East Asia	5	4/0	10; 1–13
South/Central America	2	0/2	15; 14–16
Africa	2	2/0	15; 13–16
Total	105	53/18	12; 1–16

### Clinical evidences

We considered that a definitive diagnostic characterisation was achieved in the fifty-three subjects whose neuroimaging (MRI, and/or MRV, and/or CT, and/or SPECT) was clearly reported. We tabled the neuro-ophthalmological features for each of these subjects [14,26,27,33-38,40-42,44-48,50]. Data referring to the 14 cases having the parenchymal form are detailed in Table 2. The 35 cases presenting with the non-parenchymal form are described in Table 3, and the four cases with mixed parenchymal and non-parenchymal forms are shown in Table 4. The series published by Koné-Paut and co-authors in 1998, 2002, and 2011 [32,51,52] were excluded from the tables because the patients with a neurological pattern were described as miscellaneous. The series by Uluduz and co-workers was included as a single comprehensive item, because the authors did not detail symptoms of distinct patients.

**Table 2:** **Ocular and neurological characteristics of patients with juvenile *parenchymal *Neuro-Behçet reported in the literature from 1971 to 2011**

**Patient**	**Author [Ref.]**	**OCULAR**		**NEUROLOGICAL**	
		**Signs**	**Symptoms**	**Signs**	**Symptoms**
1	Mitra [33]	Papilledema	Blurry vision Diplopia	Aseptic meningitis	Headache
				Cranial nerve palsies	
2	Vignola [34]	Retinal vasculitis	-	Aseptic meningitis	Seizure
3	Saltik [38]	Nystagmus Uveitis	Diplopia	Cranial nerve palsies	Headache
				Encephalomyelitis	
				Hemiparesis	
4	Hatachi [41]	Nystagmus Uveitis	-	Aseptic meningitis	Fever
				Cranial nerve palsies	
					Headache
					Hearing impairment
					Vertigo
					Vomiting
5	Kara [42]	Papilledema Retinal vasculitis	-	Aseptic meningitis	Fever
					Headache
					Vomiting
6	Pipitone [46]	Papillitis (bilateral)	-	Encephalomyelitis	Headache
		Retinal vasculitis			
7	Robinson [47]	Papillitis (unilateral)	Blurry vision	Encephalomyelitis	Seizure
					Vertigo
8	Metreau-Vastel [48]	-	-	Ataxia	Lapse in concentration
				Hemiparesis	
				Meningoencephalitis	
9	Metreau-Vastel [48]	-	Blurry vision	Ataxia	-
				Hemiparesis	
				Rhombencephalitis	
10	Metreau-Vastel [48]	-	-	Rhombencephalitis	Behaviour problems
					Lapse in concentration
					Loss of memory
11	Metreau-Vastel [48]	-	-	Trasverse Myelitis	Mental retardation
					Seizure
14* (3 cases)	Uluduz [14]	-	-	Relapsing Myelitis (1)	Headache (1)
					Hemiparesis (3)
					Seizure (3)

**Table 3:** **Ocular and neurological characteristics of patients with juvenile *non-parenchymal *Neuro-Behçet reported in the literature from 1971 to 2011**

**Patients**	**Author [Ref.]**	**OCULAR**		**NEUROLOGICAL**	
		**Signs**	**Symptoms**	**Signs**	**Symptoms**
1	Stern [26]	Papilledema	Diplopia	Cerebral venous thrombosis	Headache
				Cranial nerve palsies	Vomiting
2	Kerr [27]	-	-	Subarachnoid haemorrhage	Headache
					Nausea, Vomiting
					Paresthesias, bilateral facial
					weakness
3	Vignola [34]	Papilledema Retinal vasculitis	Diplopia	Cerebral vasculitis	Headache
4	Alper [35]	Papilledema	*-*	Cerebral venous thrombosis	Headache
					Nausea, Vomiting
5	Budin [36]	Papilledema Ptosis	Diplopia	Cerebral venous thrombosis	Headache
6	Chaloupka [37]	Papilledema	-	Cerebral venous thrombosis	Headache
7	Can [40]	Papilledema Uveitis	-	Cerebral venous thrombosis	Dizziness
					Headache
					Vomiting
8	Panicker [44]	Papilledema	Blurry vision Diplopia	Cerebral venous thrombosis	Headache
					Seizure
					Vomiting
9	Metreau-Vastel [48]	-	-	Cerebral venous thrombosis	-
10	Metreau-Vastel [48]	Retinal vasculitis	Blurry vision	Cerebral venous thrombosis	-
11	Metreau-Vastel [48]	Retinal vasculitis	Blurry vision	Cerebral venous thrombosis	Lapse in concentration
					Loss of memory
12	Yilmaz [50]	Papilledema	Diplopia	Cerebral venous thrombosis	Headache
					Nausea, Vomiting
35* (23 cases)	Uluduz [14]	Optic atrophy (1)	-	Cranial nerve palsies (10)	Headache (23)

**Table 4:** **Ocular and neurological characteristics of patients with juvenile *mixed parenchymal and non-parenchymal *Neuro-Behçet reported in the literature from 1971 to 2011**

**Patient**	**Author [Ref.]**	**OCULAR**		**NEUROLOGICAL**	
		**Signs**	**Symptoms**	**Signs**	**Symptoms**
1	Budin [36]	Papilledema	-	Aseptic meningitis	Headache
				Cerebral venous thrombosis	
2	Atkinson [45]	Ptosis Papilledema Uveitis	Blurry vision	Cerebral infarct	Headache, Nausea, Vomiting
				Cranial nerve palsies	
				Hemiparesis	
				Meningoencephalitis	
3	Metreau-Vastel [48]	-	-	Cerebral venous thrombosis Encephalomyelitis	-
4	Metreau-Vastel [48]	Retinal vasculitis	-	Cerebral venous thrombosis	Ataxia, Behaviour problems, Hemiparesis
				Encephalomyelitis	

In 12/53 patients (23%), neurological and/or ophthalmological symptoms anticipated any other major or minor criteria for a diagnosis of BD [14,26,27,33,34,36,40,44,47,48], and in seven additional patients, only previous oral/genital aphtae were recalled by the patient [14,35,38,41,42,45,50]. NBD was diagnosed at presentation in 13 subjects [14,36,37], and before neurological involvement in 21 patients [14,46,48]. Nine patients had a positive family history of BD (17%) [14,35,44,48], 30 patients had a negative family history [14,26,42,48], and information was not specified in 14 patients [27,34,36-38,40,41,45-47].

Forty patients (75%) complained of headache (persistent or recurrent). Related CNS involvement was specified in only 16/40 (40%) patients. Eleven of these 16 subjects (69%) had cerebral vascular involvement (thrombosis, infarct, haemorrhage, inflammation) with headache, nausea, vomiting, and/or papilledema. Nine of the 14 patients (64%) with parenchymal NBD had symptoms and signs suggestive of cerebral hemispheric involvement, including encephalomyelitis, hemiparesis, seizure, and mental changes. Two patients (14%) were reported as having the brainstem form combined with meningitis. Other syndromes were reported in four subjects: spinal cord involvement in two cases (14%), and pure acute meningeal syndrome in two cases. Detailed ophthalmological findings were clearly related to the corresponding radiological pattern in only 27 records (51%). The most frequently reported sign (14 subjects, 52%) was optic nerve head involvement, which occurred in the form of papilledema in 12 cases (86%), unilateral neuritis in one case (7%), and bilateral papillitis in another subject. Ocular inflammation was reported in 11 subjects (41%), selectively affecting the posterior pole of the eye (retinal vasculitis) in most cases (nine subjects, 82%). A disturbance of ocular motility (diplopia, nystagmus) was documented in eight subjects (30%) distributed over the three forms of the disease, and two more patients (7%) presented with ptosis. Blurry vision was a commonly reported generic symptom.

### Treatment and prognosis

An immunosuppressive therapy was reported in all patients. Different immunosuppressive drugs, mostly in combination, were used for treatment. Details on the therapy were available for 28 subjects (53%): steroids (oral and/or intravenous) were the most administered treatment (26 patients, 93%); [26,27,33-38,40-42,45-48,50] followed by colchicine (14 patients, 52%); [35,40,42,45,48,50] azathioprine (12 patients, 44%); [34,42,46-48,50] cyclosporine (five patients, 19%); [34,37,41,46,48] methotrexate (four patients, 15%); [36,41,45] anti-tumour necrosis factor drugs (three patients, 11%); [45-47] cyclophosphamide (two patients, 7%); [46,48] and chlorambucil (one patient, 4%) [27]. Adjunctive treatments consisted of anti-seizure medications, anti-coagulants, aspirin, and acetazolamide [27,34-37,42,44,48,50]. Seven patients had relapses of the neuro-ophthalmological symptoms and required renewal of therapy or implementation of ongoing immunosuppressive treatment. Nine subjects (17%) had definitive sequelae [14,48]. Motor and sensory sequelae, visual disturbances (blurry vision, low visual acuity, unilateral or bilateral blindness due to optic atrophy), personality changes with behaviour abnormalities, and cognitive difficulties were observed. One patient presenting with the vascular form died as a consequence of the rupture of a cerebral aneurysm (presence of multiple aneurysms) [27]. In the large majority of the patients, a complete recovery or significant improvement was reported [14,26,33-38,40-42,44-48,50].

In the remaining seventy-seven subjects described in the literature, neuroimaging was not available, or not clearly described (except for 2 patients with only an initial normal neuro-radiological examination reported) [12,16-25,28-32,39,43,49,51]. A distinction between parenchimal and non-parenchiaml form was conceivable for 24 patients: 19 cases (79%) presenting the parenchimal, and 5 cases presenting the non-parenchimal pattern. Ocular involvement was reported in 60 subjects (78%) but detailed in only 19 records. Among these, diplopia, cranial nerve palsies, and uveitis were the findings most frequently observed. Optic atrophy was reported in 5 patients. Concerning functional prognosis, 57% of patients showed an improvement after immunosuppressive therapy; the remaining cases (43%) had less satisfactory course due to poor responsiveness to treatment, frequent relapses, or optic atrophy.

## Discussion

BD typically affects young adults and is less common in children; this may explain why the International Study Group Criteria apply mainly to adult patient [2,53]. Furthermore, these criteria make no specific reference to CNS involvement. In the literature, the terms “paediatric” and “juvenile” are often used interchangeably. Koné-Paut [54] used the term “paediatric” for complete forms in paediatric-aged patients, and the term “juvenile” for cases where BD started prior to 16 years of age but the complete form of the disease manifested after this age.

Neurological involvement in BD was first described in adults in 1941 [55]. Some years later, histological findings were reported from the autopsy of a dermatological case [56]. The term “Neuro-Behçet syndrome” was suggested in 1954 by the Italian ophthalmologists Cavara and D’Ermo [3]. During the following decades, patients with BD have usually been labelled as having “neurological involvement**”,** as CNS involvement was considered one factor in the context of different clinical manifestations and not as a specific characterisation of the disease (i.e., NBD). A more precise correlation between neurological symptoms and anatomical lesions was made possible with progress in neuroimaging. MRI, which can be combined with angiography (MRA), represents the mainstay examination to assess the radiological patterns that distinguish the parenchymal form (more common in adult patients) from the non-parenchymal or vascular form [8,57,58]. Concerning the parenchymal involvement, MRI shows lesions mainly in the brainstem, basal ganglia, and white matter in adult patients [58-60]. The most typical MRI findings appear as lesions with high signal intensity on T2-weighted sequences [58,60-62]. A specific study on MRI interpretation of the parenchymal form in children stated that NBD should be suspected in patients who have brainstem and/or diencephalic lesions that extend along the long tracts whether or not the lesions are associated with periventricular and subcortical lesions [38]. These lesions tend to resolve on subsequent imaging studies. MRI findings have been reported as normal or abnormal in primary progressive disease with silent neurological involvement [58,62]. MRI evidence of brainstem atrophy is a well-defined feature for late NBD [58,63,64]. Concerning the non-parenchymal form, gadolinium-enhanced MRI and MRA have been used to confirm the diagnosis of CVST [65]. Compared with conventional MRI sequences, susceptibility-weighted imaging has proven to be particularly effective for detecting lesions [66]. Molecular imaging with positron emission tomography (PET) and SPECT may allow the characterisation of regional cerebral blood flow and metabolism. Similar to reports in adults, SPECT is very sensitive for detecting vascular brain involvement in paediatric patients with BD; it may support the clinical diagnosis, especially in children with negative findings on a brain structural examination, and may possibly reflect cortical diffuse low-grade inflammation better than MRI [34]. Hypoperfusion is observed mainly in the parietal lobes, basal ganglia, talami, and temporal cortex, including its mesial portion, whereas the cerebellum is the least common hypoperfused area [67]. Temporal hypoperfusion is found primarily in patients with seizures; hypoperfusion of deep grey nuclei is seen mainly in patients with different manifestations [34]. Some lesions on MRI, particularly white matter lesions, cannot be detected with SPECT, suggesting that MRI is more sensitive in this area [67]. SPECT brain perfusion imaging is performed more frequently than PET and uses different tracers [68] such as technetium-99 m hexamethylpropylene amine oxime (99mTc-HMPAO) [69], and more recently technetium-99 m ethyl cysteinate dimer (99mTc-ECD) [70,71]. Of these two tracers, 99mTc-HMPAO is limited by radiochemical instability due to its rapid decomposition *in vitro* (it must be used within 30 minutes of preparation) and by delayed imaging (40 minutes after injection) [69]. Moreover, sensitivity with 99mTc-ECD results in superior images, which allow easier interpretation of brain structures [72]. Metabolic PET imaging with [18]F-fluordeoxyglucose can identify decreased cerebral glucose consumption at the parieto-occipital cortex and brainstem in neuro-BD [73]. Thus, neuroimaging is essential to substantiate the diagnosis of NBD. In specialized and equipped centres, the ideal diagnostic workup of NBD includes molecular imaging as a complementary modality to assess real involvement of the brain, particularly considering the recent availability of hybrid technology (PET/CT and SPECT/CT) leading to increased diagnostic accuracy and performance. Improvements in imaging after effective treatment have been documented for both MRI and SPECT [34,38].

The demographics of paediatric patients with NBD (Table 1) confirm the well-known geographical distribution of the disease, with high prevalence in Western Asia and Southern Europe (particularly, France and Italy). However, reports have included all continents except for Oceania. This review found a prevalence of male cases, with a male/female ratio of 2^.^9:1. This value is definitely closer to that reported in series addressing the neurological involvement in BD independently of the age of appearance (i.e., adults and children) [3,4,8,14,50], than with reports focusing specifically on paediatric BD [29-31,49,52]. Neurological involvement appears to be strongly related to male gender. Series specifically addressing the ocular involvement of BD in childhood showed a nearly two folds prevalence of males [13,74]. The mean age at diagnosis is 11.8 years, with a median of 12 years. This means that a peak in neurological manifestations is observed around puberty, although the onset of the neurological disease may be observed after or before this age, including very early childhood.

Considering the differential diagnosis challenge and the problems related to classifying NBD, we decided to limit a detailed analysis of the neuro-ophthalmological findings to those cases with definitive radiological characterisation. More than 20% of these young patients complained of neuro-ophthalmological symptoms before the typical diagnostic elements were disclosed. This percentage rises to >35% considering the cases with a sole anamnestical report of oral and/or genital aphtae (not necessarily more than three episodes per year). This proportion is remarkably higher than that reported in other non-age-related NBD series, with no specification of whether the small number of cases with neurological onset were the youngest patients in the series [4,43]. Family history was positive in 17% of the cases. This proportion is in accordance with what Koné-Pout and co-authors referred to in 1998 as “an unexpected finding”, commenting on the high rate of familial cases in their famous survey of BD in children compared with the rate commonly reported for adult patients [32]. This evidence was further supported by a segregation analysis after stratification of BD families into paediatric and non-paediatric groups, which detected a Mendelian autosomal recessive transmission only in paediatric families. In contrast, no evidence for Mendelian inheritance was observed in patients with BD and more typical age of onset criteria [75]. Three-fourths of subjects complained of headache, often associated with other alarming neurological findings such as nausea, vomiting, or papilledema (11 of 16 detailed cases). A higher prevalence of headache characterised the patients with cerebral vascular involvement. This symptom was evidently related to the presence of intracranial hypertension secondary to vascular complications, often consisting of thrombosis within large veins and occasionally arteries. No clear distinction between tension-type and migraine headaches was expressed among the non-vascular cases. However, the prevalence of headache in children with NBD cannot be clearly stated as being higher than that in the normal population. In large surveys on primary headaches among school children in Asian and European populations, the prevalence of recurrent episodes of headache ranged from 29.1% to 87.4% [76-79].

Ocular alterations were described in 21/27 cases (78%). Optic nerve head involvement was the most frequently reported alteration (52%), chiefly in the form of papilledema. Primitive ocular involvement (i.e., excluding ocular findings secondary to neurological alterations such as papilledema, nystagmus, and extra-ocular muscle palsies) was reported in 12 cases (44%) as an inflammation of the posterior segment of the eye (posterior uveitis), with prevalence of retinal vasculitis (seven subjects). No cases had pure anterior uveitis, although iridocyclitis, with or without hypopyon, is considered a classic finding in BD [80]. Among the cases with a definitive radiological characterisation, the non-parenchymal form, in particular with CVST, was predominant. However, this evidence is influenced by a recent Turkish series; [14] excluding this series, the parenchymal and non-parenchymal forms were reported almost equally.

The prognosis was good in about 80% of cases who experienced recovery or significant improvement of neuro-ophthalmological alterations by the end of follow-up, even in cases of recurrent attacks. Nine patients (17%) had definitive visual and/or neurological sequelae. One patient presenting with a vascular form died. The rate of favourable prognosis was slightly worse (57%) in cases without radiological characterisation; this evidence is of uncertain interpretation. A possible explanation is that the lack of pathognomonic radiological features implied a delayed diagnosis and treatment. Improvement was always obtained with a slowly tapered immunosuppressive regimen, and the drugs used most often were systemic steroids, colchicine, and azathioprine, in decreasing order. The European League Against Rheumatism (EULAR) standardised operating procedures for the management of BD [81-83]. There are no controlled data to guide the management of CNS involvement in BD. For parenchymal involvement agents to be tried may include corticosteroids, interferon α (INFα), azathioprine, cyclophosphamide, methotrexate, and anti-tumour necrosis factor drugs. For dural sinus thrombosis corticosteroids are recommended. Cyclosporine A, due to its potential neurotoxicity, should be avoided in patients with BD with CNS involvement, unless necessary for intraocular inflammation. Although the effectiveness of INFα in controlling even recalcitrant manifestations of BD is well demonstrated [84,85], no patient in this review was treated with this drug, probably due to the young age. Any patient with BD and inflammatory eye disease affecting the posterior segment should be on a treatment regimen that includes azathioprine and systemic corticosteroids. Azathioprine is widely accepted as the initial agent for ocular involvement of BD. Corticosteroids rapidly suppress the inflammation but potential side effects including cataract and glaucoma cause concern. The systematic literature research considers about 20 randomized controlled trials (RCTs) on the management of patients with BD, but there are no studies relating to the management of gastrointestinal and neurological involvement. Moreover, questions related to treatment of early and pediatric form of the disease were not addressed by EULAR’s recommendations. Another problem that has not been adequately addressed in RCTs is whether anticoagulation is required for the venous thrombosis in BD.

In our experience, as well as in at least five cases reported in the literature (see Table 3), the non-parenchymal form began with binocular diplopia as predominant symptom. In these cases, diplopia was related to a peripheral ophthalmoplegia due to a “stretching” effect on the troncular pars of the sixth cranial nerve at the level of Dorello’s canal, secondary to the elevated intracranial pressure (third and fourth cranial nerve are rarely involved). However, sixth nerve palsy remains a poorly specific sign of increased intracranial pressure. Differential diagnosis should consider pathologies typically responsible for intracranial hypertension, in particular IIH or CVST due to causes other than primary vasculitis (e.g. otitis media with mastoiditis). In any case, neuroimaging is essential to support differential diagnosis of acute intracranial hypertension, focussing on exclusion of an expanding intracranial mass, CVST, IIH, or impending meningitis, with decreasing frequency.

In several cases of NBD, mostly non-parenchimal forms, early symptoms were consistent with those reported for IIH, i.e.: pulsatile headache (possibly precipitated by changes in posture), prolonged vomiting, pulsatile tinnitus, transient visual obscurations, blurred vision, and diplopia. IIH is frequently the cause of increased intracranial pressure and papilledema in young adults and, sometimes, in children. This syndrome has some characterising features, and obesity is one of the most significant [86,87]. For this reason, the Body Mass Index is an easily achievable parameter able to orient differential diagnosis in a young patient presenting with intracranial hypertension. Also a previous antibiotic regimen should be carefully investigated in children; IIH may occur after a symptom-free period of weeks following the use of different categories of antibiotics for general infections (e.g. tetracycline, or nalidixic acid). However, to reach a definitive diagnosis of IIH the revised Dandy’s criteria must be satisfied, as follows: 1) elevated cerebrospinal fluid pressure with normal composition, as documented by lumbar puncture; 2) normal neurological examination except for papilledema and possibly sixth nerve palsy; 3) no evidence of hydrocephalus or a mass, structural, or vascular lesion on MRI or contrast-enhanced CT for typical patients, or on MRI and MRA for all others; and 4) no other cause of intracranial hypertension identified [88]. Funduscopic examination of the optic nerve head is strictly recommended in all cases of suspected or confirmed elevated intracranial pressure, in order to prevent severe visual alterations related to true papilledema [89]. From a theoretical point of view, papilledema may produce an irreversible visual loss just a few weeks after intracranial hypertension has been established. Actually, the amount of axonal loss in papilledema is related to the severity and the duration of the optic disc swelling, with severity and duration acting as independent variables.

In summary, we can conclude that increased intracranial pressure, headache, papilledema, and possibly diplopia owing to the involvement of the intracranial portion of the sixth nerve represent the prevalent clinical presentations of the vascular form of NBD. Physicians should not be tempted to diagnose IIH in the case of headache with papilloedema without careful exclusion of all differential diagnoses. In children these symptoms could be early manifestations of BD and the differential diagnosis often requires multidisciplinary expertise (i.e. paediatric, neurological, ophthalmological, and immunological). With a delayed diagnosis, it may not be possible to prevent optic atrophy even with a good control of other systemic symptoms. This review updates a previous database on the topic [90] to 1989 and may assist in the interpretation of results from upcoming large cohorts of patients who were followed longitudinally [91].

## Competing interests

The authors declare that they have no competing interests.

## Authors’ contributions

PM analyzed data and wrote the manuscript; CM selected source literature; JGO conceived and supervised the study; PR helped in managing cases from personal experience; LR was responsible for radiological examination and discussion; AC was responsible for neuro-ophthalmolgical examination and discussion. All authors read and approved the final manuscript.
